# Numerical Investigation on the Ballistic Performance of Semi-Cylindrical Nacre-like Composite Shells under High-Velocity Impact

**DOI:** 10.3390/ma16103699

**Published:** 2023-05-12

**Authors:** Huiwei Yang, Dongyang Gao, Pengcheng Chen, Guoyun Lu

**Affiliations:** 1 College of Civil Engineering, Taiyuan University of Technology, Taiyuan 030024, China; yanghuiwei@tyut.edu.cn (H.Y.);; 2 Shanxi Key Laboratory of Civil Engineering Disaster Prevention and Control, Taiyuan 030024, China

**Keywords:** semi-cylindrical shells, nacre-like composites, bullet impact, residual velocity, failure mode

## Abstract

The nacre has excellent impact resistance performance, and it is attracting attention in the field of aerospace composite research. Inspired by the layered structure from nacre, semi-cylindrical nacre-like composite shells of brittle silicon carbide ceramic (SiC) and aluminum (AA5083-H116) were established. Two types of tablet arrangements (regular hexagonal and Voronoi polygons) of the composites were designed, and the same size of ceramic and aluminum shell were established for the impact resistance analyzed numerically. In order to better compare the resistance performance of the four types of structures under different impact velocity, the following parameters were analyzed including energy variation, damage characteristic, bullet residual velocity, and semi-cylindrical shell displacement. The results show that the semi-cylindrical ceramic shells have higher rigidity and ballistic limit, but the severe vibration after impact causes penetrating cracks, and the whole structure failure occurred eventually. The nacre-like composites have higher ballistic limits than semi-cylindrical aluminum shells, and the impact of bullets only causes local failure. In the same conditions, the impact resistance of regular hexagons is better than Voronoi polygons. The research analyzes the resistance characteristic of nacre-like composites and single materials, and provides a reference for the design of nacre-like structures.

## 1. Introduction

Nacre is a biomineralized material consisting of crystals and organic matrix, showing a well-organized “brick-mortar” structure [1,2], and it has received massive attention from researchers because of the high strength and toughness properties. About 95% of the nacre are made up of aragonite tablets (a brittle mineral), and the tablets are filled with a soft biopolymer organic matrix [3]. It is worth noting that nacre showed about 3–9 times more toughness than aragonite [3,4,5]. This outstanding performance is attributed to the tablet arrangement form, the tablet waves, and the multiple interfaces between tablets [6,7]. The arrangement of aragonite tablets can be divided into columnar and sheet [8]. Columnar distribution is a regular arrangement from layer to layer, and sheet distribution is a random distribution within the plane [9,10].

At present, nacre-like composites with various material combinations and structural forms have been developed [11,12,13,14,15], and many microscopic biological structural studies and dynamic analyses have also been carried out [16,17,18]. But if only the microstructure of nacre is concerned and the overall morphology is ignored, this design is clearly inadequate. To provide a protective space for the organisms inside, the pearl shell usually consists of two oval convex shell [4,19,20]. However, the study of nacre bionic design seems to focus only on planar structures, and the research of semi-cylindrical shell protective structure is worth being conducted.

The nacre-like composites have great potential to resist blast and impact due to the coexistence of multiple toughening mechanisms [21,22], and a variety of mechanical studies were conducted including drop hammer impact [21,23,24,25,26] and ballistic impact [5,27,28]. The research results show that filling soft materials between hard components can significantly improve the fatigue resistance [29] and crack resistance [30], and fully developed nacre toughening mechanisms including crack deflection, fiber pull-out, and organic matrix [31]. The sliding of aragonite and the deformation of organic matrix together form the plastic deformation of the nacre, but the main energy dissipation mechanism is the rotation and deformation of the aragonite nanograins [31,32]. Zhou and Dutta et al. studied the mechanical properties of different layered structures through parameter analysis and formula derivation, and they provide an optimization approach for the structural parameters of composite [33,34]. Stability analysis of nacre-like composites during uniaxial loading at micro and macro scales was carried out by Fabrizio et al. [35]. The conical closed-hole structure was designed, and the study of the peak acceleration during impact was carried out by Shwe et al. [36]. As a protective structure needs to face a complex external environment, so it is necessary to consider multiple forms of loading, and a single form of resistance does not adequately describe the protective performance. Therefore, the resistance mechanism and morphological damage under different impact velocity need to be clearly explained, and provide guidance for the overall performance evaluation of the structure. For different nacre-like composites, the residual velocity of the bullets and the failure process of the structure were analyzed [37,38,39,40]. From the results, the velocity or kinetic energy reduced by the bullet penetrating the composite usually demonstrated non-linear characteristics. This suggests different impact velocities resulting in different damage modes [41].

In general, the impact resistance of structure needs to be evaluated from multiple aspects. Published work has focused on nacre-like composites with planar structures, but less research has been conducted on the structure of semi-cylindrical shells. Based on elliptical convex structure of pearl shell, the semi-cylindrical nacre-like composite shells of ceramic/AA5083-H116 with two tablet arrangements forms was established, one is a regular hexagon and the other is Voronoi polygons. To compare the difference in impact resistance between brick-slurry structures and single materials, the ceramic and aluminum semi-cylindrical shells was established as a comparison object. Moreover, the parameters including energy variation, damage characteristic, bullet residual velocity and semi-cylindrical shell displacement were analyzed. Based on the above parametric analysis, the resistance characteristics of different structures were analyzed and classified. In addition, it is expected that combining the semi-cylindrical shell structure and theoretical derivation of the equivalent elastic modulus will probably provide a reference for the design of aerospace, high-speed rail, submarines, etc.

## 2. Elasticity Theoretical Analysis

### 2.1. Problem Description

**Figure 1:**
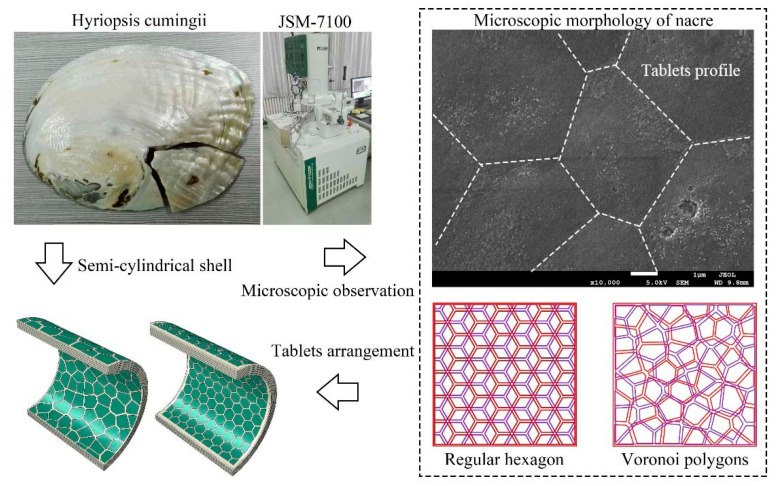
Microscopic tablets structure of nacre and finite element model.

The pearl sample of Hyriopsis cumingii from Zhejiang of China was gold-sprayed with an ion sputtering instrument (JEC-3000FC, Tokyo, Japan) and observed with a scanning electron microscope (JSM-7100, Tokyo, Japan) at an operating voltage of 5 kV, as shown in Figure 1. Based on the microscopic observation, tablets can be approximated as hexagonal and randomly distributed in the plane. In addition, the organic matrix with a mass fraction of about 5% is filled between the tablets. For this characteristic structure of the nacre, we derived the elasticity theory and performed parametric analysis.

### 2.2. Theoretical Formula Derivation

**Figure 2:**
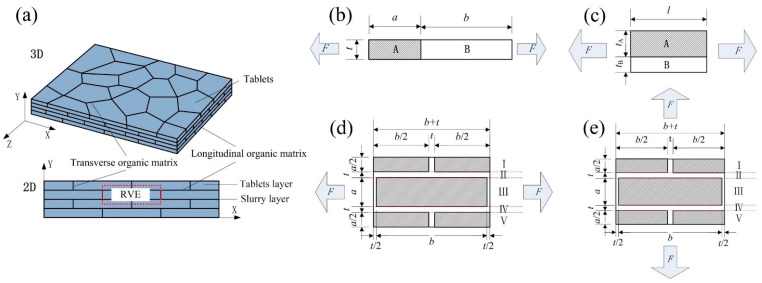
(**a**) Nacre-like composite structure model; (**b**–**e**) theoretical analysis model.

To further explain the resistance mechanism for different components of nacre-like composites, the theoretical analysis of the equivalent elastic modulus was carried out. The two-phase distribution model is used to carry out microscopic analysis, and the effect of each component on the equivalent elastic modulus can be obtained, as shown in Figure 2a. The tensile stiffness of the homogeneous elastomer is known [42]:

(1) $$K=\frac{EA}{l}$$

The elastomers in Reuss connection mode are shown in Figure 2b. When they are subjected to a load F and the effect of Poisson’s ratio is not considered, the tablets and the slurry are subjected to the same stresses at both sides. The following relationship is satisfied:

(2) $$\left\{\begin{array}{l} u=u_{A}+u_{B}\\ F=\frac{E_{i}t}{a+b}u=\frac{E_{A}t}{a}u_{A}=\frac{E_{B}t}{b}u_{B} \end{array}\right.$$

Combining the two equations above and simplifying:

(3) $$\frac{a+b}{E_{i}t}=\frac{a}{E_{A}t}+\frac{b}{E_{B}t}$$

Reuss relationship exists for tensile stiffness, and the total series stiffness can be obtained:

(4) $$\frac{1}{K}=\frac{1}{K_{A}}+\frac{1}{K_{B}}$$

(5) $$K=\frac{E_{A}E_{B}t}{bE_{A}+aE_{B}}$$

The equivalent modulus of elasticity is obtained from Equation (3), and it is rewritten as:

(6) $$E_{i}=\frac{E_{A}E_{B}(a+b)}{bE_{A}+aE_{B}}$$

(7) $$\frac{1}{E_{i}}=\frac{1}{a+b}\left(\frac{a}{E_{A}}+\frac{b}{E_{B}}\right)$$

Define dimensionless size (fraction of volume occupied) $\Phi_{1}=\frac{a}{a+b}$, then Equation (7) can be rewritten as [43]:

(8) $$\frac{1}{E_{i}}=\frac{\Phi_{1}}{E_{A}}+\frac{1-\Phi_{1}}{E_{B}}$$

The elastomers in Voigt connection mode are shown in Figure 2c. When they are subjected to a load F and the effect of Poisson’s ratio is not considered, the tablets and the slurry are subjected to the same strain, $u=u_{A}=u_{B}$. The following relationship is satisfied:

(9) $$\left\{\begin{array}{l} F=F_{A}+F_{B}\\ F=\frac{E_{i}(t_{A}+t_{B})}{l}u,\;F_{A}=\frac{E_{A}t_{A}}{l}u_{A},\;F_{B}=\frac{E_{B}t_{B}}{l}u_{B} \end{array}\right.$$

where F, $F_{A}$, and $F_{B}$ represent the combined forces on the sides of the elastomers, the combined forces on the sides of elastomer A and elastomer B, respectively. Based on the coordinated displacement equation $u=u_{a}=u_{b}$, the following can be obtained from Equation (9):

(10) $$\frac{E_{i}(t_{A}+t_{B})}{l}=\frac{E_{A}t_{A}}{l}+\frac{E_{B}t_{B}}{l}$$

Therefore, the tensile stiffness of the Voigt mode satisfies the parallel relationship, and the equivalent elastic modulus can be obtained:

(11) $$K=K_{A}+K_{B}$$

(12) $$E_{i}=\frac{E_{A}t_{A}+E_{B}t_{B}}{\left(t_{A}+t_{B}\right)}$$

Define dimensionless size (fraction of volume occupied) $\Phi_{2}=\frac{t_{A}}{t_{A}+t_{B}}$, Equation (12) can be rewritten as:

(13) $$E_{i}=E_{A}\Phi_{2}+E_{B}(1-\Phi_{2})$$

Figure 2d shows the elastomers under loading in the longitudinal direction, where the tablets length is set as b and the width is set as a. It is assumed that the organic matrix width and thickness between the tablets are t. The representative volume unit can be divided into five parts, each part has a series relationship inside, and the elastic modulus of each part according to Equation (8) can be expressed as:

(14) $$\left\{\begin{array}{l} \frac{1}{E_{I}}=\frac{1}{E_{\mathrm{III}}}=\frac{1}{E_{V}}=\frac{\Phi_{1}}{E_{p}}+\frac{1-\Phi_{1}}{E_{m}}\\ E_{\mathrm{II}}=E_{\mathrm{IV}}=E_{m} \end{array}\right.$$

where $\Phi_{1}{}^{\prime }=\frac{b}{b+t}$, which means the volume fraction of tablets in the layer, $E_{p}$ and $E_{m}$ denote the elastic modulus of the tablets and slurry, respectively. When small strains occur in composites, the shear stresses between the five parts can be disregarded and parallel relationships exist between the five regions. Equivalent elastic modulus in the longitudinal direction can be obtained according to Equation (13):

(15) $$E_{i}=\Phi_{2}\frac{E_{p}E_{m}}{\Phi_{1}E_{m}+(1-\Phi_{1})E_{p}}+(1-\Phi_{2})E_{m}$$

where $\Phi_{2}{}^{\prime }=\frac{a}{a+t}$ is the volume fraction of the tablets in the composites. As shown in Figure 2e, when loading in the transverse axis direction, the parallel relationship exists within each part, and the elastic modulus of each part can be obtained based on Equation (13):

(16) $$\left\{\begin{array}{l} E_{I}=E_{\mathrm{III}}=E_{V}=\Phi_{1}E_{p}+(1-\Phi_{1})E_{m}\\ E_{\mathrm{II}}=E_{\mathrm{IV}}=E_{m} \end{array}\right.$$

The series relation exists between the five parts, and the equivalent elastic modulus in the transverse axis direction can be obtained according to Equation (8):

(17) $$\frac{1}{E_{i}}=\frac{\Phi_{2}}{\Phi_{1}E_{p}+(1-\Phi_{1})E_{m}}+\frac{1-\Phi_{2}}{E_{m}}$$

This equation can be rewritten as:

(18) $$E_{i}=E_{m}\frac{\Phi_{1}E_{p}+(1-\Phi_{1})E_{m}}{\left[\Phi_{2}+(1-\Phi_{1})(1-\Phi_{2})\right]E_{m}+\Phi_{1}(1-\Phi_{2})E_{p}}$$

From the above theoretical analysis, it can be seen that the nacre-like composites exhibit different equivalent elastic modulus in the longitudinal and transverse directions. Increasing the volume fraction of the tablets can increase the equivalent elastic modulus in the longitudinal direction. However, for the transverse axis direction, the same trend is shown only when the elastic modulus of the tablets is much higher than the slurry. In addition, increasing the elastic modulus of both the tablets and the slurry can increase the equivalent elastic modulus of the composites, but in most cases increasing the elastic modulus of the tablet has a greater effect. Analysis from the design perspective, increasing the volume fraction of tablets as much as possible and using materials with higher elastic modulus can effectively reduce the elastic deformation of the composites and improve the impact resistance of the structure.

### 2.3. Validation of Theoretical Results

**Figure 3:**
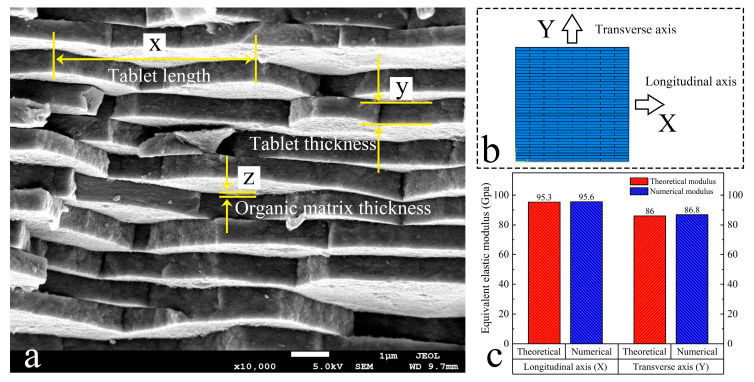
(**a**) Dimension of cross-sectional microstructure of nacre; (**b**) numerical geometry model; (**c**) comparison of theoretical and numerical in different direction.

Two-dimensional nacre-like composites were simulated using a linear elastic model. As shown in Figure 3a, the nacre-like composites model is scaled based on the measured dimensions, a=0.48 mm, b=4.98 mm, and z=0.02 mm. The total size of the model is 20 mm × 20 mm, and a displacement constraint of 0.2 mm is applied for the X and Y directions, respectively. Based on the study by Katti et al. [44], the elastic modulus of aragonite tables $E_{p}=\;100\;\mathrm{Gpa}$, the elastic modulus of the organic matrix $E_{m}=20\mathrm{Gpa}$, and Poisson’s ratio are set to 0.3. Quasi-static analysis was performed by the Abaqus/standard, and the two-dimensional nacre-like composites model is shown in Figure 3b.

Based on the results of finite element calculation, the equivalent elastic modulus is compared with the theoretical model, as shown in Figure 3c. In the X direction, the difference between the theoretical model and the numerical model is about 0.3%. In the Y direction, the difference between the theoretical model and the numerical model is about 0.9%. The equivalent elastic modulus of the theoretical and numerical models is generally consistent in both directions, indicating that the theoretical results can be used for evaluating the equivalent elastic modulus of the nacre-like composites.

### 2.4. Theoretical Parameter Analysis

**Figure 4:**
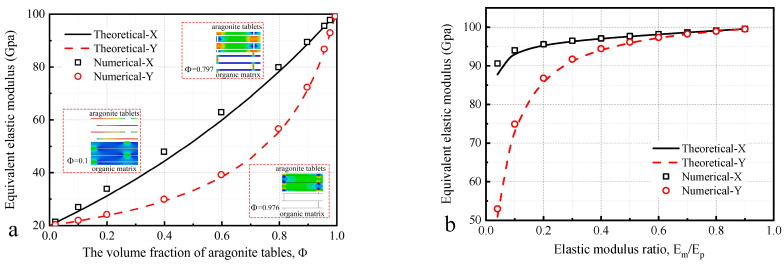
Equivalent elastic modulus with (**a**) volume fraction of tables and stress diagram; (**b**) elastic modulus ratio of slurry and tables, $E_{m}/E_{p}$.

Figure 4a shows the effect of different volume fraction of tables on the equivalent elastic modulus, where the volume fraction of tables is controlled by the thickness of the different components. With the volume fraction of aragonite tablets increasing, the equivalent elastic modulus increases in both X and Y directions. When Φ=0, the equivalent elastic modulus of the composite is 20 Gpa, and the structure consists of only the slurry. When Φ=1, the equivalent elastic modulus of the composite is 100 Gpa, and the structure consists of only the tablets. Because of the difference of structural form in X and Y direction, when 0≤Φ≤0.7, the equivalent elastic modulus increases more in the X direction than Y direction. When 0.7≤Φ≤1, the equivalent elastic modulus increases more in the Y direction than X direction. When the volume fraction of the tablet is greater than 90%, which means that the volume fraction of the slurry is less than 10%, better isotropic properties and equivalent elastic modulus can be achieved.

Figure 4b shows the effect of elastic modulus ratio (slurry and tables, $E_{m}/E_{p}$) on the equivalent elastic modulus, and the model dimensions remain the same as the model in Section 2.3. When $E_{m}/E_{p}>0.2$, the difference in equivalent elastic modulus in X and Y directions is less than 10%, which is close to homogeneous material. When $0.04<E_{m}/E_{p}<0.2$, the equivalent elastic modulus decreases more significantly in the Y direction than X direction. When $E_{m}/E_{p}=0.04$, the equivalent elastic modulus in X direction is 87.6 Gpa, and it is 50.9 Gpa in Y direction. The difference between X and Y direction is 72.1%. Combining Figure 4a,b, the equivalent elastic modulus of the nacre-like composites is greater in the X direction than Y direction in most cases.

From the perspective of design, when the elastic modulus of the slurry is greater than 20% of the tablets, better isotropic properties and equivalent elastic modulus can be achieved.

## 3. Materials and Numerical Methods

### 3.1. The Generation of Nacre-like Composites

As shown in Figure 1, inspired by the elliptical convex structure of pearl shells, we established a semi-cylindrical nacre-like composite shells. Based on the arrangement form of the tablets under the microscopic observation, the regular hexagon and Voronoi polygon structures were established respectively. In addition, the ceramic and aluminum semi-cylindrical shells were established with the same dimensions, and the comparative analysis of the impact resistance for the four structures was carried out.

The radius of the semi-cylindrical composite shells is 50 mm and the height is 100 mm. The hard tablets are divided into five layers, each layer thickness is 1 mm. The interlayer is filled with a slurry of 0.5 mm thickness, and the slurry thickness between tablets in the same layer is about 2 mm. The slurry and tablet are connected to each other by tied constraints. The hard tablets in adjacent layers are staggered, and the distance between the center of the tablets is 11.8 mm. The staggered arrangement of tablets can be divided into two forms: one is the regular structure, it is assumed that the tablets are in positive hexagonal. The other is irregular structure, the tablets are considered to be Voronoi polygons, and two semi-cylindrical composite shell were modeled by Rhino 7.0.

**Figure 5:**
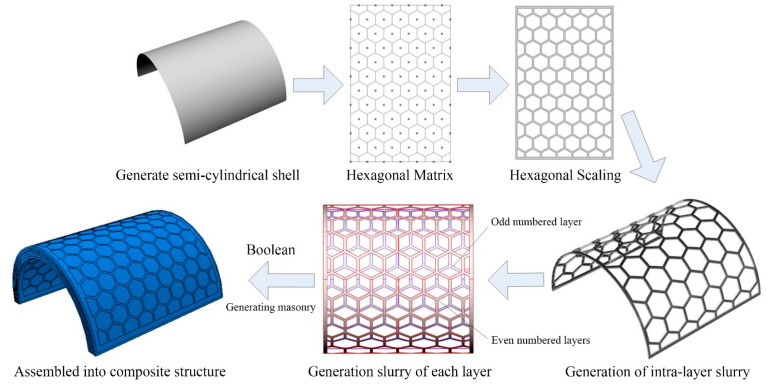
Generation of the nacre-like structure with hexagonal shape.

The model of the regular structure is constructed by parametric modeling method, and the side length of the hexagon is 9 mm. The model is formed by hexagonal array, and the detailed modeling process is shown in Figure 5.

**Figure 6:**
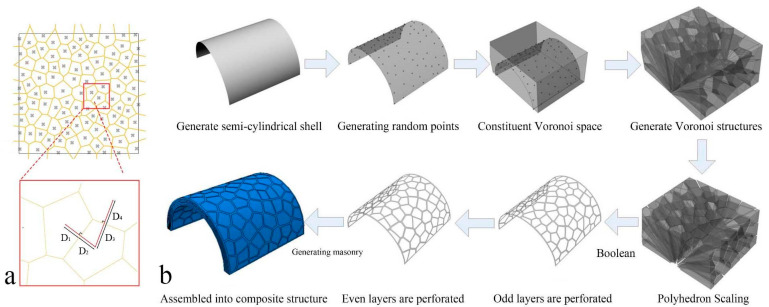
(**a**) The 2D Voronoi polygon structure; (**b**) generation of the nacre-like structure with Voronoi polygons.

The irregular polygon structure model is constructed using the Voronoi algorithm. As shown in Figure 6a, the connection lines of any two adjacent points are perpendicular bisector for their common boundary line, which means $D_{1}=D_{2},\;D_{3}=D_{4}$ [45].

The isometric transformation is used to project the Voronoi polygon in space onto the semi-cylindrical shell [46,47,48]. The geometric dimensions were the same as the regular hexagon, and the generation process is shown in Figure 6b.

### 3.2. Materials Models

#### 3.2.1. Material Model of Ceramic

**Figure 7:**
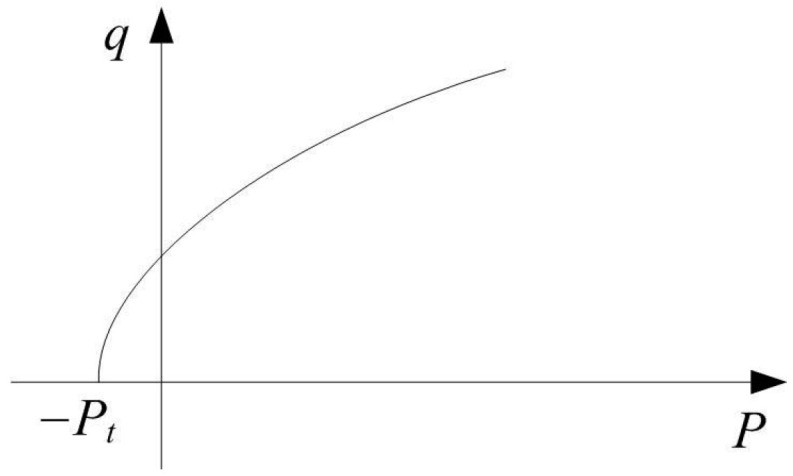
Exponent form of Drucker–Prager plastic model.

The Drucker–Prager plastic model in exponential form describes the strength of the ceramic, as shown in Figure 7. The exponent form can be expressed as follows:

(19) $$F=a_{i}q^{b}-P-P_{t}$$

Combined with the Drucker–Prager plastic model, the Johson–Cook rate-related model, and the Mie–Grüneisen state equation and the JH-2 model is rewritten to describe the material properties of ceramic [49]. The above material parameters are summarized and are listed in Table 1

The initial ductile damage criterion was used for the ceramic, and criterion requires the functional relationship between equivalent plastic strain and stress triaxiality [50,51]. The stress triaxiality can be written as [52]:

(20) $$-\frac{P}{\sigma_{i}}=-P/\left[\sigma_{\mathrm{HEL}}A_{i}{\left(\frac{P}{P_{\mathrm{HEL}}}+\frac{T}{P_{\mathrm{HEL}}}\right)}^{N}\right]$$

where $a_{i}=P_{HEL}/{\left(A\sigma_{HEL}\right)}^{1/N}$ and $b_{i}=1/N$. After obtaining the material parameters $a_{i}$ and $b_{i}$, the uniaxial compression yield stress $\sigma_{c}$ can be calibrated by the following equation.

(21) $$P_{t}=a_{i}\sigma_{c}^{b}-\frac{\sigma_{c}}{3}=T$$

The plastic damage of the JH-2 model at constant pressure is defined as:

(22) $${\bar{\varepsilon }}_{f}^{pl}=D_{1}{\left(P^{*}+T^{*}\right)}^{D_{2}}\;\mathrm{when}\;{\bar{\varepsilon }}_{f,\min}^{pl}\leq {\bar{\varepsilon }}_{f}^{pl}\leq {\bar{\varepsilon }}_{f,\max}^{pl}$$

where $P^{*}=P/\sigma_{i}^{\max}$ and $T^{*}=T/\sigma_{i}^{\max}$. The equivalent plastic strain at the beginning of the damage is known, and the following pressure relations can be obtained according to the damage evolution relations of the JH-2 model.

(23) $$P=P_{\mathrm{HEL}}{\left(\frac{{\bar{\varepsilon }}_{f}^{pl}}{D_{1}}\right)}^{1/D_{2}}-T$$

The relationship between the equivalent plastic strain ${\bar{\varepsilon }}_{f}^{pl}$ and the stress triaxiality is achieved by the expression, and the damage data of ceramic are shown in Table 2.

The Mie–Grüneisen state equation is used to describe the hydrodynamic behavior, and the pressure is expressed as:

(24) $$P=\frac{\rho_{0}c_{0}^{2}\eta }{{\left(1-s\eta \right)}^{2}}$$

where $\eta =1-\rho_{0}/\rho =\mu /(1+\mu )$, $K_{1}=\rho_{0}c_{0}^{2}$ and $K_{2}=\rho_{0}c_{0}^{2}(2s-1)$ is the pressure density relation for JH-2 model. Through a quadratic polynomial, the parameters $c_{0}$ and s are identified.

#### 3.2.2. Material Model of Aluminum (AA5083-H116)

The slurry medium is simulated with aluminum (AA5083-H116), and the material model includes Johnson–Cook constitutive law [53] and Johnson–Cook fracture criterion [54]. Johnson–Cook constitutive law is a special Mises plastic model, which is parsed in the form of hardening criteria and rate correlation. The model is suitable for deformation at high strain rates, especially for transient dynamic analysis. The Johnson–Cook plastic model uses a Mises yield face containing the flow law to describe the plastic reinforcement phase, and the static yield stress $\sigma^{0}$ is defined as:

(25) $$\sigma^{0}=\left[A_{j}+B{\left({\bar{\varepsilon }}^{pl}\right)}^{n}\right]\left(1-{\hat{\theta }}^{m}\right)$$

where $\hat{\theta }$ is a dimensionless temperature, it is defined as:

(26) $$\hat{\theta }\equiv \left\{\begin{array}{c} 0\\ \left(\theta -\theta_{t}\right)/\left(\theta_{m}-\theta_{t}\right)\\ 1 \end{array}\right.\begin{array}{c} for\\ for\\ fot \end{array}\begin{array}{c} \theta <\theta_{t}\\ \;\theta_{t}\leq \theta \leq \theta_{m}\;\\ \theta >\theta_{m} \end{array}$$

Johnson–Cook fracture criterion is based on a liner damage accumulation rule, and the element is considered to be invalid when the damage parameter exceeds 1. The damage parameter ω is defined as:

(27) $$\omega =\sum \left(\frac{\Delta {\bar{\varepsilon }}^{pl}}{{\bar{\varepsilon }}_{f}^{pl}}\right)$$

where the failure strain is assumed to be dependent on a dimensionless plastic strain rate, $\frac{{\dot{\bar{\varepsilon }}}^{pl}}{{\dot{\varepsilon }}_{0}}$; $\hat{\theta }$ is defined earlier in the Johnson–Cook hardening model. The dependencies are assumed to be separable in the following form.

(28) $${\bar{\varepsilon }}_{f}^{pl}=\left[d_{1}+d_{2}\exp\left(d_{3}\frac{p}{q}\right)\right]\left[1+d_{4}\ln\left(\frac{{\dot{\bar{\varepsilon }}}^{pl}}{{\dot{\varepsilon }}_{0}}\right)\right]\left(1+d_{5}\hat{\theta }\right)$$

where the intrinsic dissipation of heat can be calculated by inelastic heat fraction η, and the η is assumed to be constant and typically about 0.9 for metals [55]. In this paper, the slurry medium is defined by aluminum AA5083-H116 [56], the material properties are shown in Table 3.

#### 3.2.3. Aluminum Material Model Validation

The Johnson–Cook material model was validated by referring to the perforation experiments of aluminum armor plate by Borvik et al. [58]. Racht–Ipson model is used to fit the residual velocity curve [59], its formula is as follow:

(29) $$V_{r}=a^{\prime }{\left(V_{i}^{P}-V_{bl}^{P}\right)}^{1/P}$$

**Figure 8:**
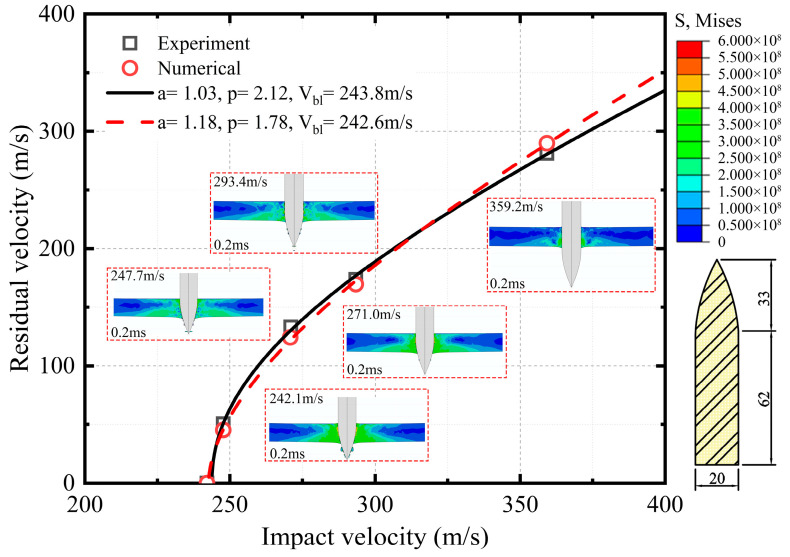
Comparison between experimental and numerical for the residual velocity of bullet impact AA5083-H116 [58].

Based on bullet perforation experiments, the axisymmetric algorithm was used to build a circular plate with a diameter of 500 mm and a thickness of 20 mm. The local stress plots of the bullet penetrating the aluminum plate at different impact velocities are captured, and the bullet geometry is shown in Figure 8. The comparison shows that the maximum residual velocity difference between numerical and experiment is 10.4% at the impact velocity of 247.7 m/s, and the difference is lower as the impact velocity increases. The numerical calculations show agreement with the experiments, indicating that the aluminum material model is reliable.

## 4. Resistance Characteristic Analysis

### 4.1. Impact Response of Semi-Cylindrical Shells

In order to compare and analyze the impact resistance of the nacre-like composites and the single material, the same dimensions of ceramic and aluminum were chosen to analyze by Abaqus/explicit. The mass of the bullet is 14.82 g and the initial velocity is 500 m/s. The bullet is constrained to be rigid body, and is a cylinder with radius 5 mm and length 10 mm. In this paper the unit of stress is Pa.

**Figure 9:**
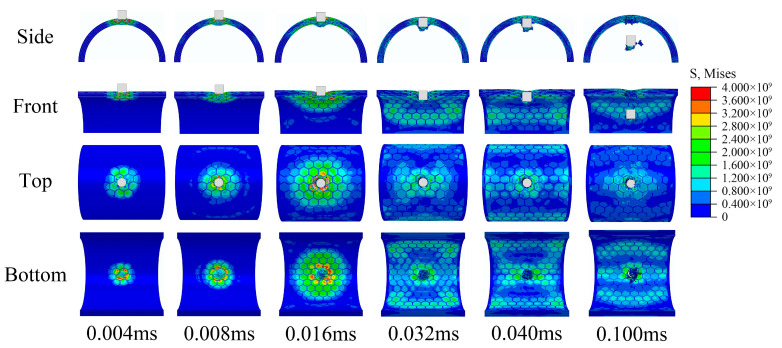
Impact response process of regular composite.

**Figure 10:**
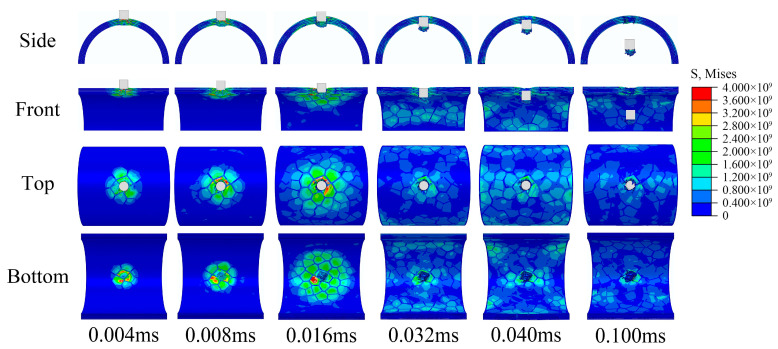
Impact response process of irregular composite.

The impact response of the semi-cylindrical composite shells is observed from different directions, as shown in Figure 9 and Figure 10. Two different distribution forms of the semi-cylindrical shells are penetrated by the bullet, and the penetration process can be divided into four stages:

1. The bullet impacts the composite outer surface, and the outer surface is concaved.
2. Tablets were impacted to fragments, and some tablets fall off from the inner surface of composites.
3. Tablets almost completely show failure at the impact position, and the slurry begins to deform and show failure.
4. The slurry shows failure completely and the composites are penetrated by bullets.

**Figure 11:**
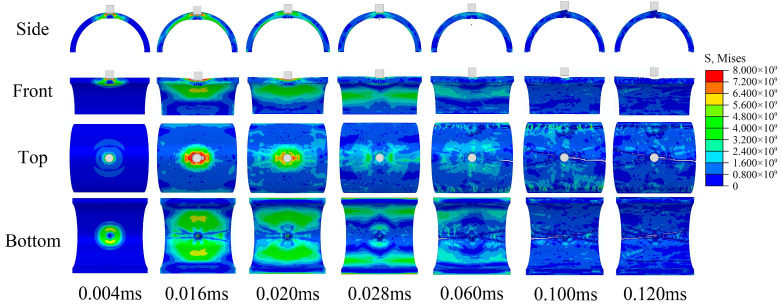
Impact response process of semi-cylindrical ceramic shell.

**Figure 12:**
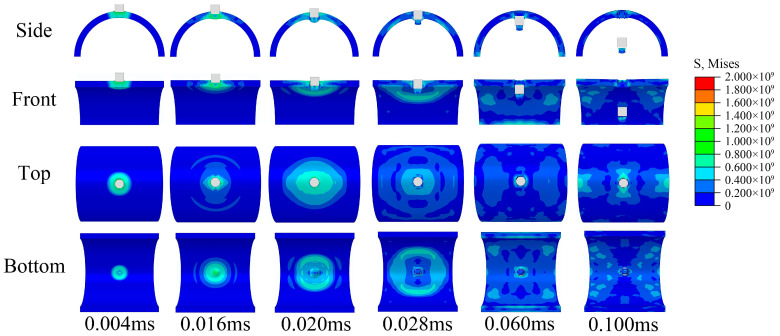
Impact response process of semi-cylindrical aluminum shell.

As shown in Figure 11, the bullet reaches its maximum intrusion depth and the ceramic inner surface begins to indicate failure at 0.02 ms. The crack then gradually expands and eventually forms penetrating cracks, and the ceramic eventually shows failures completely. As shown in Figure 12, the aluminum shell is damaged by the shear of the bullet edge and produces only a large conical metal fragment in front of the bullet.

The stress diffusion for the composite is more extensive and uniform than ceramic, and no penetration cracks were observed in the waist of the semi-cylindrical composite shells. This may be because the layered nacre-like structure effectively prevents the concentration of stress and slows the propagation of stress waves. From the results, the ceramic was damaged as a whole and completely lost the ability to resist subsequent impacts, while the composites were only locally damaged and still maintained the ability to resist subsequent impacts. From the resistance results, there are two possible reasons why penetrating ceramic requires higher impact velocity:

1. Ceramic produce more morphological damage during resistance and absorb more impact energy of the bullet.
2. The semi-cylindrical shell structure weakens the brittle fracture of the ceramic and enhances the overall resistance.

In addition, localized fracture forms are different for ceramic and composites. The ceramic produces a large number of fragments splashing inwards after breaking. However, due to the connection of the internal slurry, the composite produced only one large fragment in front of the bullet. For some fragile internal protection objects, composite structures may produce better effect. For both regular and irregular forms of tablets distribution, the regular form produced better impact resistance, this is probably because the regular distribution is more conducive to the diffusion and propagation of stress waves.

### 4.2. Energy Variation Analysis

**Figure 13:**
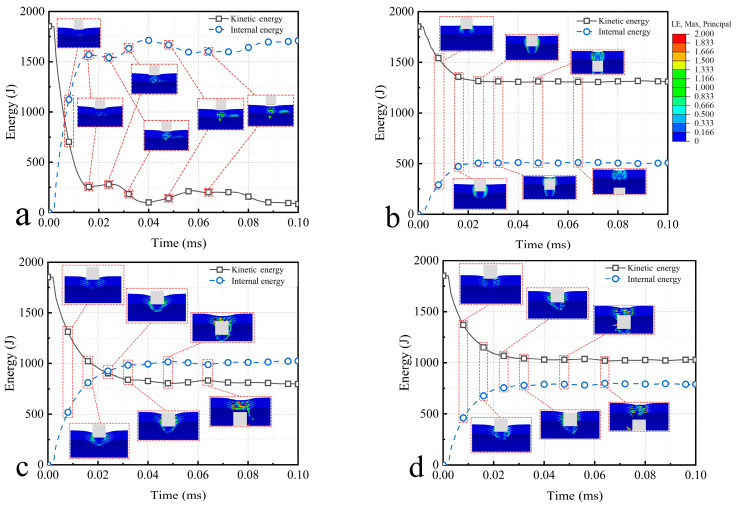
Energy variation and strain diagram of (**a**) ceramic; (**b**) aluminum; (**c**) regular composite; (**d**) irregular composite.

The system energy is analyzed, and the main focus is on the variation of the kinetic energy and the internal energy, as shown in Figure 13. The initial kinetic energy of the bullet is 1852.6 J, and the initial distance between bullet and semi-cylindrical shell is 1 mm. The conversion of the kinetic energy and the internal energy occurs mainly before 0.02 ms, and the rate of energy conversion decreases with time. This indicates that the resistance of the semi-cylindrical shell decreases as the depth of bullet penetration increases. It is worth noting that the kinetic energy and internal energy does not change monotonically after 0.02 ms, but fluctuate within a certain range. This further suggests the accumulation and release of energy in the process of impact resistance, which in macroscopic terms means that the semi-cylindrical shell is subjected to the impact and undergoes violent vibration. In addition, ceramics have larger energy fluctuations than composites and aluminum, this suggests that the ceramic vibrates more intensely. The local strain of the bullet impact process is shown in Figure 13.

## 5. Discussion of Impact Velocity

### 5.1. Damage Characteristics Analysis

**Figure 14:**
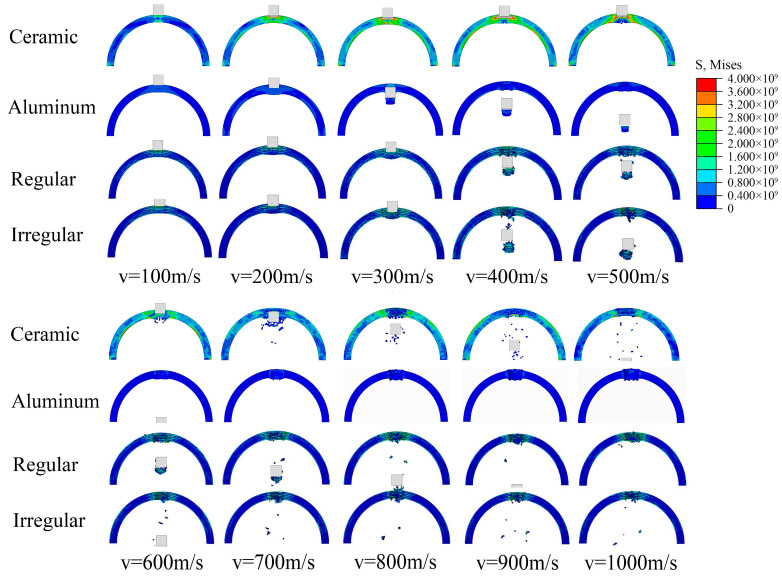
Side view of semi-cylindrical shells under different velocity impact.

The dynamic response of semi-cylindrical shells at bullet impact velocities from 100 to 1000 m/s was simulated, and their deformation and damage characteristics was analyzed. Take the side view for observation, as shown in Figure 14.

Based on the deformation and damage process of the semi-cylindrical shells, the damage can be classified from slight to severe into the following five types. (A) Partial deformation (bullets are bounced); (B) the bullet did not penetrate the shell, but the structure produced fragments; (C) bullet penetrates the shell, but does not produce large amounts of fragments; (D) bullets penetrate the shell, generating a large amount of high-speed fragments; (E) failure of the whole structure.

For semi-cylindrical aluminum shells, when the impact velocity is less than 200 m/s, the bullet is bounced, which can be considered as type A damage. When the impact velocity is greater than 200 m/s, the aluminum shell is penetrated and a large fragment is produced, which can be considered as type D damage. When the bullet velocity is less than 300 m/s, ceramics and composites produce the type A damage. When the bullet velocity is greater than 400 m/s and less than 600 m/s, the composites produce the type C damage. When the bullet velocity is greater than 600 m/s, the composite produce the type D damage. However, for ceramic structures, when the bullet velocity is greater than 400 m/s and less than 500 m/s, the structure is not penetrated and a large number of fragments are produced, which can be considered to belong to type B damage. When the bullet velocity is greater than 500 m/s, the structure has already developed penetrating cracks, so it belongs to type E damage. In addition, the fragments produced by the ceramics and composites increase with the increase in the impact velocity of the bullet, this indicates that the higher impact energy causes more fragmentary to the semi-cylindrical shell structure.

### 5.2. Residual Velocity Analysis

**Figure 15:**
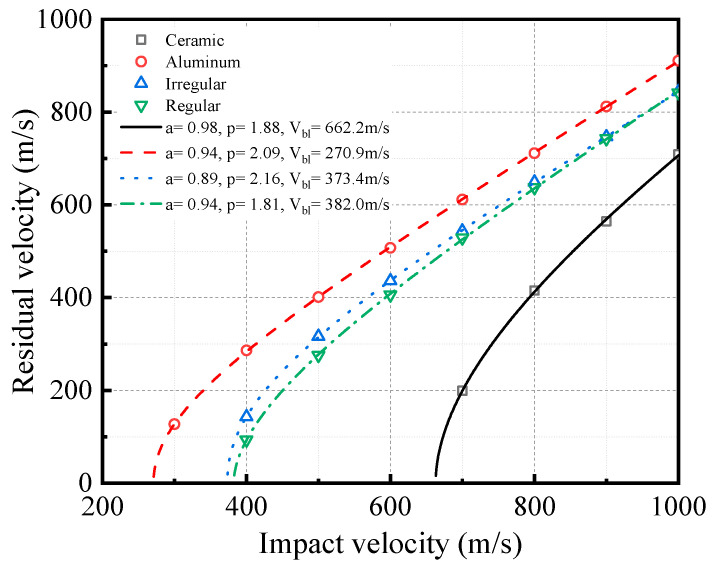
Residual velocity of bullet impacting semi-cylindrical shells.

As can be seen in Figure 15, aluminum shell has the lowest ballistic limit of 270.9 m/s, and the ballistic limit of the aluminum shell is about 39.5% lower than the composite. The ballistic limits of the composites are about 382.0 m/s and 373.4 m/s, respectively, and the ballistic limit of ceramic shell is about 662.2 m/s. The ballistic limit of ceramics is about 73.4% higher than the composites. However, the difference between ceramics and composites decreases as the bullet impact velocity increases. When the bullet impact velocity is 1000 m/s, the residual velocity of the composites is 18.7% higher than ceramic. In addition, the residual velocity of the irregular structure is slightly higher than the regular structure, but the difference decreases with increasing bullet impact velocity, and they are almost consistent at 1000 m/s.

### 5.3. Semi-Cylindrical Shell Top Displacement Analysis

**Figure 16:**
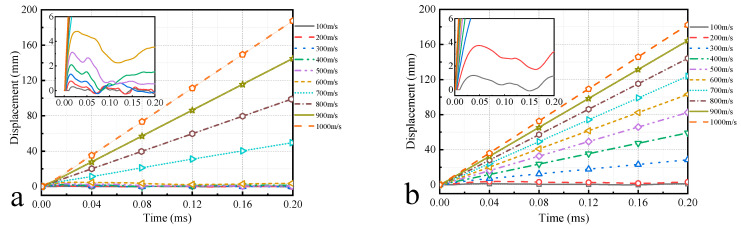

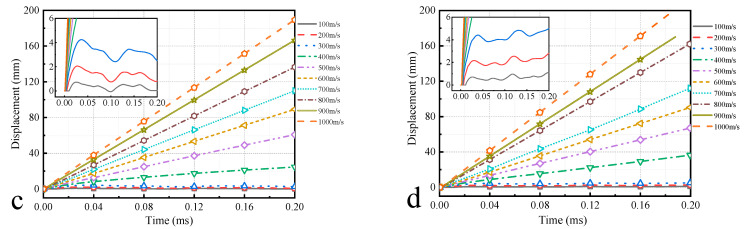
Displacement-time curve at the top center of semi-cylindrical shells. (**a**) Ceramic; (**b**) aluminum; (**c**) regular composites; (**d**) irregular composites. Curves of the same color represent the same impact velocity.

The displacement at the top center of semi-cylindrical shells was compared and analyzed, as shown in Figure 16. When the impact velocity is high, the displacement of the top center increases linearly with time, which indicates the bullet penetrated the semi-cylindrical shell, and the part impacted by the bullet flew away from the semi-cylindrical shell. When the impact velocity is low, the displacement of the top center of the semi-cylindrical shells is kept to fluctuate within a certain range, which indicates that the bullet is bounced and the semi-cylindrical shells vibrate under the impact. When the bullet is bounded, the vibration amplitude of the semi-cylindrical shells increases with the increase in the impact velocity of bullet.

## 6. Summary and Conclusions

Inspired by the microstructure of the nacre, two types of nacre-like composites of different tablet arrangements were created, and the equivalent elastic modulus of nacre-like composites has been theoretically derived. Referring to the morphology of the pearl shell, four types of semi-cylindrical protective structure was established to study the impact resistance. Morphological damage of the semi-cylindrical shells and the residual velocity of the bullets were analyzed, the damage formed to the structure are divided into five types. In addition, parametric analysis of bullet impact velocity was carried out. Based on the above research process, the following conclusions can be drawn.

1. Based on the two-dimensional structure of the nacre, an elastic theoretical model of the two-phase distribution was established. The theoretical model was verified to be reliable and can be used to predict the equivalent elastic modulus of nacre-like composites.
2. From the theoretical results and numerical models, increasing the elastic modulus of the tablets can enhance the equivalent elastic modulus of the nacre-like composite more effectively. When the elastic modulus of the slurry is greater than 20% of the tablets, and its volume fraction is less than 10%, the isotropic properties of the composite can be better achieved.
3. The semi-cylindrical ceramic shell has the highest stiffness and ballistic limit, but the violent vibration generated after the impact causes penetrating cracks. The cracks eventually lead to the whole failure of the structure. The nacre-like composites have higher ballistic limit than aluminum shell. The bullet impact only causes local failure, and the structural integrity is better maintained. Semi-cylindrical aluminum shells have the lowest ballistic limit, it was damage by the shear of bullet’s edge and forms a large fragment only in front of the warhead.
4. Under the same conditions, the regularly composites have less damage and lower bullet residual velocity than the irregularly composites, this indicates regular structure has better impact resistance performance.
5. The semi-cylindrical shell vibrated under the bullet impact, and the vibration amplitude increased with the velocity of bullet impact. When the bullet impact velocity is high, the vibration of ceramic is more intense than nacre-like composite and aluminum.

## Figures and Tables

**Table 1:** Material parameters of ceramic.

Material Properties	Value
Density ρ (kg/m^3^)	3215
shear modulus, G (Gpa)	193
Drucker-Prager plastic model	
The constant of Drucker-Prager model, $A_{i}$	0.00392
The constant of Drucker-Prager model, $b_{i}$	1.5384
Hardening parameters of static water pressure, $P_{t}$ (Gpa)	0.75
Non-axial compressive yield stress, $\sigma_{c}$ (Mpa)	6605.66
Johson-cook rate-related models	
Reference strain rate, ${\dot{\varepsilon }}_{0}$ (s^−1^)	1
Strain hardening coefficient, C	0.009
Mie-Grüneisen state equation	
Bulk speed of sound, $c_{0}$ (m/s)	8272.2
Slope of the linear, s	1.32

**Table 2:** Damage parameters of ceramic.

Equivalent Plastic Strain, ${\bar{\varepsilon }}_{f}^{pl}$	Stress Triaxiality
0.0000757409	14.9582
0.000387262	5.81024
0.0010059	3.31439
0.00198007	2.20507
0.00334828	1.59126
0.00514314	1.20551
0.00739319	0.941806
0.0101241	0.75036
0.0133591	0.604923
0.0171197	0.490442
0.087533	−0.0269666
0.227363	−0.235092
0.447555	−0.373262
0.756814	−0.483609
1.16251	−0.579447
1.67109	−0.666391
2.28835	−0.74727
3.01956	−0.823696
3.86958	−0.896673

**Table 3:** Johnson–Cook model parameters of AA5083-H116 [45,57].

Material Properties	Value
Density ρ (kg/m^3^)	2750
Young’s modulus of elasticity, E (Gpa)	70
Poisson’s ratio, ν	0.3
Inelastic heat fraction, η	0.9
Specific heat, $C_{p}$ (J/kgK)	910
Proof/Yield stress, $A_{j}$ (Mpa)	215
Strain hardening coefficient, B (Mpa)	280
Strain hardening coefficient, n	0.404
Strain hardening coefficient, C	0.0085
Thermal softening constant, m	0.859
Reference strain rate, ${\dot{\varepsilon }}_{0}$ (s^−1^)	0.001
Reference temperature, $\theta_{r}$ (K)	293
Melting temperature $\theta_{m}$ (K)	893
$d_{1}$	0.096
$d_{2}$	0.049
$d_{3}$	3.465
$d_{4}$	0.016
$d_{5}$	1.099

## Data Availability

Not applicable.

## Glossary

K	Tensile stiffness
A	Cross-sectional area
l	Length
a,b,t	Structure size
u	Strain
$E_{i}$	Equivalent elastic modulus
$K_{A},K_{B}$	Tensile stiffness of A, B elastomers
$u_{A},u_{B}$	Strain of A, B elastomers
$E_{A},E_{B}$	Young’s elastic modulus of A, B elastomers
$\Phi_{1}$	Fraction of volume occupied, a/(a+b)
F	Load
$F_{A},F_{B}$	Load of A, B elastomers
$\Phi_{2}$	Fraction of volume occupied, $t_{A}/(t_{A}+t_{B})$
$E_{I},E_{\mathrm{II}},E_{\mathrm{III}},E_{\mathrm{IV}},E_{V}$	Elastic modulus of the series part
$\Phi_{1}{}^{\prime }$	Volume fraction of tablets in the layer, b/(b+t)
$E_{m}$	Elastic modulus of slurry
$E_{p}$	Elastic modulus of tablets
$\Phi_{2}{}^{\prime }$	Volume fraction of the tablets in the composites, a/(a+t)
Φ	Volume fraction of the tablets
T	Hydrostatic pressure
N	the constant of Drucker-Prager model
$P_{\mathrm{HEL}}$	The pressure at the Hugoniot elastic limit (HEL)
$\sigma_{\mathrm{HEL}}$	The equivalent stress at the HEL
${\bar{\varepsilon }}_{f}^{pl}$	The strain at failure
$a_{i},b_{i},A_{i},D_{1},D_{2},\sigma_{i}$	Material constants
$\rho_{0}$	Ceramic density
μ	Volumetric strain
$\sigma^{0}$	Static yield stress
${\bar{\varepsilon }}^{pl}$	Equivalent plastic strain
$\hat{\theta }$	Dimensionless temperature
θ	Current temperature
$\theta_{m}$	Melting temperature
$\theta_{t}$	Transition temperature
$\theta_{r}$	Reference temperature
ω	Damage parameter
$\Delta {\bar{\varepsilon }}^{pl}$	Increment of the equivalent plastic strain
$d_{1}-d_{5}$	Material parameters determined from different mechanical test
${\dot{\varepsilon }}_{0}$	Reference strain rate
η	Inelastic heat fraction
$c_{0}$	Bulk speed of sound
s	Slope of the linear
$A_{j}$	Proof/Yield stress
B,C,n	Strain hardening coefficient
$\sigma_{c}$	Non-axial compressive yield stress
G	Shear modulus
ρ	Density
E	Young’s modulus of elasticity
ν	Poisson’s ratio
$C_{p}$	Specific heat
m	Thermal softening constant
$a^{\prime },P$	Constants of best fit data points
$V_{bl}$	Ballistic limit;
V	Impact velocity of bullet
$V_{r}$	Residual velocity of bullet
